# Neuroscience, Empathy, and Violent Crime in an Incarcerated Population: A Narrative Review

**DOI:** 10.3389/fpsyg.2021.694212

**Published:** 2021-07-28

**Authors:** Valeria Saladino, Hannah Lin, Elisa Zamparelli, Valeria Verrastro

**Affiliations:** ^1^Department of Human Sciences, Society and Health, University of Cassino and Southern Lazio, Cassino, Italy; ^2^Department of Biological Sciences, Columbia University, New York, NY, United States; ^3^Institute for the Study of Psychotherapies, Rome, Italy; ^4^Department of Medical and Surgical Sciences, University “Magna Graecia” of Catanzaro, Catanzaro, Italy

**Keywords:** empathy, deviance, violent crimes, neuroscience, psychopathy (PSY)

## Abstract

Empathy is a fundamental construct that allows individuals to perceive and understand the cognitive and emotional state of others. Empathy is not only a psychological and sociological concept; it also heavily impacts our daily lives by affecting our decisions and actions. Empathy is connected to and involves specific parts of the brain which, if damaged or of reduced volume, can lead to actions that are morally unjust, aggressive, or simply denoting a lack of understanding and sensitivity. The literature affirms that the low level of empathy, guilt, embarrassment, and moral reasoning displayed by violent and psychopathic criminals is strongly associated with empathy-linked brain regions that are smaller in size or less developed. The aim of this review is to show empirical data over the last 5 years on the connection between empathy and neuroscience among violent and psychopathic offenders, reflecting on future research on the topic.

## Empathy and Neuroscience

### Definition of Empathy

The construct of empathy does not have a universally recognized definition, as previous studies have focused on philosophical and behavioral aspects (Batson, 2009; Bernhardt and Singer, 2012). Lately, research has focused on identifying the underlying neural network processes. Neuroscience has made an important contribution to understanding the neural basis of empathy. A recent definition states that the empathic process occurs when the observation or imagination of affective states of others induces shared feelings in the observer and involves various components such as affective sharing, self-awareness, and self-other differentiation (Singer and Lamm, 2009). Empathy contributes to the development of positive social interactions and helps us to understand and react to others’ behaviors.

However, empathy is not automatic or obligatory. Indeed, our reaction to others’ feelings derive from a series of factors such as the situation, the empathizer, beliefs, and goals compared with emotions of others. For instance, the suffering of strangers might not affect us because we are not motivated or interested to be involved in their feelings; however, if we attend to the suffering of friends or family, empathy activation changes. An example is a reaction to the cry of a child; some people might get annoyed, others might put themselves in the shoes of the embarrassed parent, still others might put themselves in the shoes of the suffering child, and our empathic activation changes whether that child is our grandson or a stranger.

This wide range of reactions depends on intrapersonal and situational characteristics. Also, our capacity to understand others’ feelings is not necessarily connected with a prosocial attitude. Empathy must be regulated. Excessive empathy or lack of empathy denotes an inability of individuals to adapt to situations. Compared to animals, humans have greater cognitive abilities. For example, humans can process the emotional states of others using the theory of mind (ToM) (Stone, 2006), as defined by the developmental psychology. This evolutionary aspect could lead to extraordinary prosocial actions, such as caring for individuals of different species, and to the worst actions, like violence and dehumanization. There are some individuals, such as those who commit violent crimes and those with psychopathy who often have empathy deficits. Thus, a better understanding of the component of empathy and its neurodevelopment is needed.

### Evolution and Types of Empathy

According to developmental and social psychology, empathy is the affective response derived from the understanding of others’ feelings. First, empathy manifestations occur during childhood among infants (6-months-old), who prefer altruistic characters rather than not- cooperative ones (Hamlin et al., 2007). Children of 18–25 months tend to sympathize with others in the absence of emotional stimuli, experimenting with some form of affective perspective-taking (Vaish et al., 2009). Moreover, prosocial behaviors emerge at 12 months, a period in which children can care for people who need help (Warneken and Tomasello, 2009).

From a clinical and neurobiological point of view, empathy can be differentiated into affective empathy and cognitive empathy (Saladino et al., 2020a). Affective empathy is defined as the ability to understand and share the emotional experiences of others through an autonomic response, while cognitive empathy is defined as the ability to understand and share the point of view of others, allowing inferences on mental or emotional states (Cox et al., 2011). Affective empathy is involuntary, and it develops early than the cognitive empathy. It relates to the somato-sensorimotor response, such as the feeling of distress experiment by children when another child is crying (Dondi et al., 1999). The cognitive component of empathy is related to the ToM (the ability to interpret the mental state of others, their thoughts, and beliefs), the executive function of attention, memory, and self-regulation. Both ToM and self-regulation are associated with the functioning of the prefrontal cortex (medial and dorsolateral regions) and of the subcortical limbic structures (Zelazo et al., 2008). The prefrontal cortex continues to develop during adolescence and adulthood. This area of the brain is also responsible for controlling one’s emotions and actions (Diamond, 2002).

Empathy involves several areas of the brain that are not limited to the cortex but also include the autonomic nervous system (ASN), the hypothalamic-pituitary-adrenal axis (HPA), and the endocrine system, associated with the regulation of emotional and bodily states. According to the components of both cognitive and affective empathy, scientists (Decety and Jackson, 2004; Decety, 2005, 2007; Decety and Meyer, 2008) identified two models of processing empathy: a bottom-up model associated with the affective component and a top-down model linked to the cognitive component. The bottom-up processing is mediated by the amygdala, hypothalamus, and orbitofrontal cortex (OFC) for the affective arousal; top-down processing is associated with the anterior insula (AI), medial prefrontal cortex mPFC, and ventromedial prefrontal cortex (vmPFC) for emotion awareness, and OFC, mPFC, and dorsolateral prefrontal cortex (dlPFC) for emotion regulation, control of emotion and motivation. Both processing models are connected and influenced by the ASN and the endocrine system (Decety, 2011).

### Empathic Circuit and Core Network of Empathy

The research on neural substrates that are involved in the empathy circuit was guided by the hypothesis which states that there is a shared network of regions involved in both the empathic experience and the first-person affective experience (Gallese and Goldman, 1998; Preston and de Waal, 2002). Mostly, neuroimaging studies on empathy have been focused on the perception of pain. Data from two meta-analyses on the topic showed the involvement of some specific brain areas, such as the AI, the posterior-anterior (pACC), and the anterior medial cingulate cortex (aMCC). These regions are implicated in several functions, such as pain processing, evaluation and perception of emotions, and interoceptive awareness (Kober et al., 2008; Craig, 2009; Duerden and Albanese, 2011; Lindquist et al., 2012).

In supporting the shared-networks hypothesis, recent studies with fMRI have shown that pACC and aMCC are associated with both observing and feeling pain and relate with the AI, which integrates the cognitive and affective information of the personal perception of pain (Allman et al., 2010; Shackman et al., 2011). Altogether, these areas contribute to the integration of the information to the global emotional state, leading to a modulation of behavioral response, which is part of the empathic response (Singer and Lamm, 2009). Other evidence of the involvement of the AI in empathy shared networks derived from a study in participants with alexithymia. Among these people, the AI is less activated when they try to understand their own emotions and when they are empathizing with others’ pain (Silani et al., 2008; Bird et al., 2010). Empathic response and the associated core network can occur through a stimulus-response or perception-based condition, in which the subject is exposed to the presence of a concrete visual stimulus and reacts through empathic activation, or through an inference-based condition, in which individuals are exposed to abstract cues and are influenced by their perspective-taking skill and their previous experiences in attributing emotional states of others (Amodio and Frith, 2006; Frith and Frith, 2006). The activation of the core empathic-related networks in the inference-based condition is associated with ToM and mentalization’s networks, characterized by the vmPF, the superior temporal sulcus (STS), the temporoparietal junction (TPJ), the posterior cingulate cortex (PCC). The perception-based condition is related to action observation areas, as dorsolateral (dlPFC) and dorsomedial (dmPFC), and the inferior parietal cortex (IPC). The reciprocal interaction between these two routes of empathy allows for a complete representation of others’ emotional states (Danziger et al., 2009; Zaki et al., 2009).

As mentioned before, empathic activation is modulated by individual and contextual characteristics. Beliefs and the personal interpretation of the context influence the empathy process and the activation of the empathic core network. For instance, scientific evidence found less activation in AI when individuals witnessed the suffering of others if the subject of empathy had committed actions considered amoral by the empathizer or was perceived as part of the outgroup (Molenberghs et al., 2016; Molenberghs and Louis, 2018). The reduction in the AI area is also associated with increased activation of ventral striatum/nucleus accumbens (NAcc), linked to reward and desire for revenge (de Quervain et al., 2004; Cohen et al., 2009; Takahashi et al., 2009). Thus, empathy can be solicited through AI activation, associated with help, understanding, and prosocial behaviors, and counteracted by the activation of the NAcc and the antagonistic motivational system. Moreover, according to some research on the topic, the individual can generate and control empathic responses. When the empathizer takes the perspective of friends or family, there is increased activation of the AI and midcingulate areas, associated with higher empathic response. When the empathizer takes the perspective of a stranger, there is a decreased connection between the AI and TPJ.

Thus, the individual can increase or decrease their empathic response, using perspective-taking as a strategy based on personal engagement with the subject of empathy.

### Brain Lesions and Empathy

Considering the number of areas involved in the empathic process, it is necessary to consider several disorders derived from brain lesions or dysfunctional development. Moreover, as emphasized by cited studies on the affective and cognitive components of empathy, a person could have an issue related to affective empathy but not to cognitive empathy, such as in psychopathy, schizophrenia, depersonalization, and narcissistic personality disorder (Kumari et al., 2009; Schiffer et al., 2017; Nascivera et al., 2019). Meanwhile, autism spectrum disorder, for instance, is characterized by a deficit in cognitive empathy and not in affective empathy (Baron-Cohen, 2011; Fan et al., 2014; Saladino et al., 2020d).

Studies on brain injury have provided neurobiological information related to the dissociation between affective empathy and cognitive empathy. Specifically, bilateral damage to the amygdala can compromise affective empathy (Hurlemann et al., 2010). The amygdala, together with the hypothalamus, hippocampus, and OFC, are fundamental for affective arousal and automatic discrimination of a stimulus. The reciprocal interaction between the amygdala, OFC, and STS leads to the processing of affective signals. Damage in the mPFC, associated with emotion awareness, can compromise cognitive empathy (Shamay-Tsoory et al., 2009). Emotive understanding can overlap with ToM and mentalization and is also associated with AI and vmPFC, which, together with mPFC, integrate cognitive and emotional understanding, creating a balanced condition represented throughout the PFC.

Findings on degenerative neurological diseases support this distinction between the affective and cognitive components of empathy. Snowden et al. (2003) and Nathani et al. (2020) analyzed the empathy process among patients with Huntington’s disease (HD) and frontotemporal dementia (FTD), both associated with social cognitive deficits. They found the same poor empathy conditions but patients with HD reported more affective empathy deficits, while FTD patients reported cognitive empathy issues. These results can be attributed to a deficit in the ToM for FTD and a deficit in interpreting social situations for HD.

Also, Adolphs et al. (2000) studied the role of the somatosensory cortex in emotion processing. They found that damage in the right somatosensory cortex can compromise the capacity to recognize facial expressions. In fact, during the processing of facial expressions, we rely on the representations of the somatosensory cortex. However, there are conflicting opinions about mutual influence between the first-person experience of emotion and recognition of the same emotions in others. Data from a study on patients with bilateral facial paralysis found that they reported no deficit in emotion recognition, even if they did not express facial emotions (Keillor et al., 2002).

In a review on brain-imaging studies among violent offenders over the last 10 years (Bogerts et al., 2018) authors found deviations in structure and deficit in function among PFC, OFC, AI, as well as in temporolimbic structures such as amygdala, hippocampus, and parahippocampus, areas important in both affective and cognitive empathy, and in control of impulsive and aggressive behaviors (especially hypothalamus and limbic system).

Finally, clinical evidence showed that damage in PFC can provoke a deficit in empathy and interpersonal behaviors. Studies on patients with neurological lesions reported reduced empathy, especially if the damage involved the right hemisphere and the ventromedial region. Also, patients who have lesions in medial/cingulate PFC have a deficit in social interactions and emotional interpretation. These patients present a sense of apathy, poor concentration, and interest in the environment. These results suggest a key role of PFC in the empathy process and perspective-taking skills (Shamay-Tsoory et al., 2003, 2005).

Empathy is important in managing behaviors and understanding the emotional states of others. The association between empathic deficit, neuroscience, violent crime, and psychopathic personality has therefore received increasing attention from the scientific community.

## Psychopathy and Zero Degrees of Empathy

### The Construct of Psychopathy

Psychopathy has been conceptualized according to several definitions and characteristics: sometimes as an antecedent of violence and crime, other times as a hereditary and biological condition that affects social and empathetic skills. Despite the large number of conceptualizations, theories, and research on the subject, psychopathy remains a concept to be investigated (Skeem et al., 2011). Psychopathy was identified as a mental disorder characterized by antisocial and morally reprehensible behavior and the commission of crimes with no apparent sense of guilt, shame, or remorse. Psychopaths exhibit a generalized lack of empathy with both family members and strangers, which does not allow them to differentiate emotional stimuli from impulses. This mechanism leads these subjects to equate love and sexual excitement, sadness, frustration, anger, and irritability. The concepts of psychopathy and antisocial personality are connected; specifically, antisocial personality represents the attempt to transpose psychopathy on a more operational and concrete level. Psychopathy can be considered the malignant form of antisocial personality because the behaviors of psychopaths are predatory, programmed, destructive, indifferent to the consequences, and without remorse (Fornari, 2012).

Psychopaths are divided into two categories: passive and aggressive. Passive psychopaths are parasitic toward others and exploit them. Often, they may have trouble with the law but manage not to suffer serious consequences and punishments. Psychopaths of this type mostly commit what are referred to as “white-collar crimes,” i.e., economic crimes that do not involve the use of threats and physical violence. Aggressive psychopaths, on the other hand, commit serious crimes; especially those who are characterized by sexual sadism can commit serial murders of a sexual nature and, at the basis of their crimes, there seems to be the need for continuous stimulation provided by sexual arousal. The two main traits that distinguish psychopathic behavior are the inability to feel a normal degree of empathy and affection toward other people and the repeated implementation of antisocial behaviors.

A further classification of psychopathy is that of primary and secondary. The first one is characterized by self-overestimation, the tendency to use violence as a tool or means to reach a goal, pathological lying, superficial charm, lack of emotions, low levels of empathy and remorse, manipulative attitudes, low levels of stress or anxiety. Primary psychopathy does not necessarily involve the commission of crimes. Secondary psychopathy is more related to crime and deviance, with tendencies toward impulsivity and delinquency. This type does not necessarily refer to an emotional or empathic deficit, unlike primary psychopathy. Thus, the subject could develop empathy or an attachment toward someone, but morality, irresponsibility, and violence are distinctive elements in relating to others. In this case, we are faced with subjects with marked impulsiveness and a tendency to violence, linked to the aggression or anger of the subject.

According to Caretti and Craparo (2010), psychopathy is a deviant developmental disorder, characterized by a condition of instinctual aggression and the inability to have a mutual relationship. In this disorder, the emotional and behavioral elements are emptied of human feeling. The psychopathic personality includes four areas (Glenn et al., 2009): (a) interpersonal area (manipulation, pathological lying, high self-esteem); (b) affective area (absence of remorse, numbness, lack of empathy); (c) lifestyle area (search for strong feelings, impulsiveness, selfishness); and (d) antisocial area (poor behavioral control, delinquency, and violence). The element that characterizes the psychopathic personality seems to be the low level of empathy that results in the inability to identify with others and in the almost total indifference to the harmful consequences of one’s violent or criminal actions (Lavazza and Sammicheli, 2012). This is a condition that tends to persist for the whole lifespan. These characteristics are revealed from the early years of childhood and manifest themselves in both genders, although males seem to be more prone to aggressive attitudes. One of the most accredited theories about psychopathy and its relationship with empathy is that this disorder derives from an empathic deficit and dysfunction of the responses of sensitivity and social identification. These characteristics can lead to violent and aggressive attitudes (Harris et al., 2001). Although the use of violence and impulsivity appears to be a prerogative of psychopathy, as shown by several media cases such as those of Ted Bundy, John Wayne Gacy, and Dennis Rader, many of them are organized, have “cold blood” in social relations, have a high capacity of decision-making, and are not violent or impulsive. Thus, some psychopaths could show ability in planning and organizing a crime (Lilienfeld and Arkowitz, 2007).

Robert Hare (1991) studied and classified the concept of psychopathy, stating that psychopathy has a hereditary predisposition (Hare, 1999). In order to scientifically evaluate the disorder, Hare structured a questionnaire called the Psychopathy Checklist-Revised (PCL-R) (Hare, 2003). PCL-R provides a reliable assessment of the psychopathy construct in a wide range of settings and for clinical and research purposes, but its elective application is in the assessment of psychopathy in criminals and forensic psychiatric patients. The tool is administered mainly by psychologists and psychiatrists, but its results are also used by all professionals working in the judicial, penitentiary, and forensic fields who find themselves evaluating and comparing psychiatric expertise in the field of criminal proceedings. The PCL-R consists of 20 items to which a score (0, 1, 2) must be attributed after the file review and the interview. The items are divided into 4 components which converge into 2 factors: Factor 1. Interpersonal / Affective: Describes the interpersonal and affective traits of social interaction, investigating the selfish, callous and remorseless use of others. It is divided into Interpersonal (Component 1) and Affective (Component 2); Factor 2. Social deviance: investigates the unstable and antisocial lifestyle, mainly regarding aspects of impulsiveness, irresponsibility, lack of scruples, and measures aspects related to criminal behavior. It is divided into the Lifestyle components (Component 3) and Antisocial (Component 4). By administering the questionnaire to the US prison population, researchers found that an elevate percentage of prisoners reached or exceeded the threshold level for the diagnosis of psychopathy (Ibidem; Lavazza and Sammicheli, 2012). These results were confirmed by further studies which showed how psychopathy is widespread especially among the prison population (Coid et al., 2009; León-Mayer et al., 2015).

### Psychopathy, Empathy, and Neuroscience

Many studies have collected data with the aim of investigating the link between psychopathy, neuroscience, and empathy.

Gregory et al. (2012) investigated differences in structural gray matter by comparing a group of violent offenders with antisocial disorder and psychopathy, violent offenders with antisocial disorder, and healthy non-offenders. Gray matter has a role in empathic processing, moral judgment, and prosocial behaviors. Results from structural magnetic resonance imaging and volumetric voxel-based morphometry showed that the first and the second group have a reduced gray matter volume in both the anterior rostral prefrontal cortex (Brodmann area 10), important in higher cognitive functions, such as memory, judgment, or problem solving (Burgess et al., 2007), and in temporal poles (Brodmann area 20/38), which are involved in linguistic processes, language comprehension, and production (Ardila et al., 2014). This evidence confirmed that there are brain differences between violent offenders with and without psychopathy and healthy non-offenders.

Fazel and Danesh conducted a study in 2002 showing that 47% of the male population in prison and 21% of the female population suffer from antisocial personality disorders. Following these statistics, about 25% of inmates fall within the psychopathic diagnostic criteria (Lilienfeld and Arkowitz, 2007). In a survey of British inmates, it was found that 7.7% of men and 1.9% of women suffered from psychopathy (Coid et al., 2009). Murders are the most frequent crime found in psychopaths (93.3%), both in “cold blood” and premeditated. The percentage drops significantly for non-psychopaths (48.4%), who are more likely to commit a homicide for a passional reason (Woodworth and Porter, 2002). Indeed, psychopaths mostly use instrumental violence, rather than reactive violence, diffused among other offenders. The main difference is that instrumental violence, also defined as proactive and predatory violence, is purpose-driven, controlled, and cognitively mediated, while reactive violence is emotion-mediated and could derive from a provocation or uncontrolled rage. Individuals who use instrumental violence are less likely to be involved in the criminal justice system thanks to their capacity to methodically plan and organize crimes. On the contrary, individuals who use reactive violence have more difficulties in hiding their crimes, because they react following an impulse (Chase et al., 2001; Woodworth and Porter, 2002).

The model proposed by Hare seems to apply only to forensic populations (Debowska et al., 2017) for the inclusion of antisocial conduct. However, psychopathy can lead to both criminal and non-criminal paths. For instance, a higher percentage of psychopathic traits were found in a corporate sample (Babiak et al., 2010; Hassall et al., 2015). Also, the most appreciated presidential performances in U.S. were those carried out by presidents with high psychopathic traits (Lilienfeld et al., 2012). Thus, according to Boduszek and Debowska (2016), criminality and violence just partially represent psychopathy. They established a different model for psychopathy, the Psychopathic Personality Traits Model (PPTM). They (Boduszek et al., 2017) considered psychopaths as individuals who have low affective responsiveness and empathy. These characteristics lead to callous traits and difficulty in response to others’ emotions, low cognitive responsiveness, cognitive empathy and mentalization ability, high interpersonal manipulation and sense of grandiosity, and egocentricity. The authors identify two elements that most characterize psychopathy, self-love, self-centeredness, and cognition. Indeed, even if psychopaths present difficulties in both affective and cognitive empathy, a recent study on justice-involved psychopaths individuals demonstrated that they understand the cognition and beliefs of others and have a deficit in processing the affective state and emotional words (Intrator et al., 1997; Shamay-Tsoory et al., 2010). These findings need to be interpreted also considering the intelligence quotient (IQ), which could moderate the relationship between psychopathy and emotional responding. Individuals with high traits of psychopathy and higher IQ are more likely to correctly adapt their social response desirably. The cognitive empathy and responsiveness in this case can be a contingent feature of psychopathy or derive from the level of IQ (Boduszek et al., 2017).

In their study, Boduszek et al. (2017) found that psychopathy should be evaluated in a continuum across its main components. The behavior and the tendency to commit a specific crime change based on the level of psychopathy. According to this definition, they identified different groups: low psychopathy, moderate affective/cognitive responsiveness, high interpersonal manipulation, moderate psychopathy, and high psychopathy group. For instance, individuals with high interpersonal manipulation and egocentricity and low affective and cognitive responsiveness are more likely to commit property offenses and white-collar crime than the low psychopathy group. The high psychopathy group represents only 7.1% of the prison population, showing results in contrast with most theorizations on psychopathy, and dispelling the myth that inmates are mostly psychopaths.

Another author who contributed to defining the relationship between empathy, violence, and psychopathy is Simon Baron-Cohen. In “The Science of Evil” (2011), he developed some fundamental assumptions on the relationship between empathy, neuroscience, and violence, specifically psychopathy. Using the neurobiological concept of empathy, Simon Baron-Cohen theorized the possibility of tracing actions traditionally defined as “evil” to an empathic defect. He pointed out that empathy should not be treated as a binary variable–that is, following the criterion of presence/absence–but through a spectrum of increasing degrees. Baron-Cohen theorized seven levels (0 to 6). In this regard, a test on the Empathy Quotient (EQ) was developed by the scientist’s research group for adults and even children (through a specially modified version). The author defined psychopathic disorder as a “zero-negative” disorder, corresponding to grade 0 on the theorized empathy scale, involving a tendency to paying constant attention to oneself, incapacity in understanding others’ behavior and emotions, and a consequent negligent or aggressive act. Thus, a zero-negative degree of empathy determines a potential to harm others due to a substantial inability to understand the real consequences of one’s actions. In line with recent literature, Baron-Cohen assumed that the level of empathy is attributable to the “Empathic Circuit.” Relying on modern fMRI techniques, he found that it was possible to have a clear idea of the brain areas involved in empathic behavior. Baron-Cohen identified areas involved in recognition and processing of others’ emotions and adequate responses, such as the inferior parietal lobule and furrow (both, significantly, areas included in the system of mirror neurons), the middle cingulate cortex (MCC), AI, middle prefrontal cortex, the frontal orbital cortex (OFC), tempo parietal junction (TPJ), the superior temporal sulcus (STS) and the amygdala. As previously documented in the introduction, these brain areas are not to be considered as part of some sort of linear chain, but as a brain network having multiple connections. The author highlighted that the correct functioning of this circuit is substantially responsible for the empathy. In accordance with this, it is the connection between regions of the brain that lead to violence, not the single regions themselves (Hirschtritt et al., 2018).

Other neurological and neuroscientific studies underline the importance of those areas in the empathic process. De Oliveira-Souza et al. (2008) found a reduced volume of gray matter in specific areas identified as the “moral brain,” involved in moral decisions. These regions are the medial prefrontal cortex, the superior temporal sulcus, and the anterior temporal cortex. Blair et al. (2005) hypothesized that psychopathy might be generated by an early-onset amygdala dysfunction that compromises the processing of negative affect and therefore moral socialization. Individuals with this dysfunction would not be able to associate moral transgression with people’s suffering or to correctly judge fear-evoking statements (Marsh and Cardinale, 2014). Blair et al. (2005) also underline the role of the orbitofrontal and ventrolateral cortex in the selection of responses and self-control.

Kiehl (2006) proposes a complementary hypothesis that shifts attention to the “emotional brain.” According to the author, psychopathy derives from a disorder of the paralimbic system that causes an anatomical reduction and a lower level of activation in emotional learning and decision-making. This system includes the septum, the amygdala, the subcortical areas (involved in the regulation of emotional responses), the hippocampal areas (related to memory), and the cortical areas (involved in social interactions). The same author also conducted a study with fMRI (Kiehl et al., 2001) focused on the emotional deficit. They found reduced activity in the brain areas important in the acquisition of emotional responses–the amygdala, the anterior and ventral dorsal cingulate cortex, the posterior cingulate, and the ventral striatum–when psychopaths were placed in front of images or words with an emotional impact.

The latest line of the investigation was reported by Malatesti and McMillan (2010) and is linked to the “social brain.” Specifically, regarding the processing of facial expressions, the authors noted that psychopaths have reduced activity in the fusiform gyrus when they observe facial expressions that express fear (Deeley et al., 2006), sadness, and happiness (Blair et al., 2001; Dolan and Fullam, 2006; Hastings et al., 2008; Dadds et al., 2009).

The research presented shows that that psychopathy is a complex construct that is yet to be defined.

## Dysfunctional Violent Brain

Our brain allows us to speak, move, and feel emotions. According to recent neuroscientific theories, it could therefore also influence any violent behavior. Raine (2013) elaborated on this possible connection in a study involving forty-one violent crime prisoners in California. Equipped with an escort, handcuffs, and chains, the detainees were subjected to a CT scan. Their brains were also examined with positron emission tomography (PET) scans, allowing the examination of the metabolic activity of major brain regions, such as the prefrontal cortex.

The Continuous Performance Test (Rosvold et al., 1956) was also used to activate this area. The test consisted of pressing a button every time the image of an “O” was projected on a computer for 32 min, without interruptions. For this task, it was essential to maintain high concentration over a long period. After this test, the participants underwent PET, which measured the glucose levels reached during the previous experiment. An increase in glucose metabolism in the PFC corresponded to higher activation during the task. From the analysis of the control group, which involved forty-one men of the same age, it was revealed that in the experimental group, there was a lack of activation in the prefrontal cortex. Furthermore, the experimental group also showed a reduction in prefrontal glucose metabolism compared to the control group. Therefore, it seems that the activation of the prefrontal cortex plays an important role in the violent behavior of an individual (Raine, 2013).

The prefrontal cortex acts on violent behavior based on five different levels:

(1) On an emotional level, a malfunction of this region of the brain could compromise the management of control over the most primitive parts such as the limbic system, which generates primary and instinctive emotions like anger. On the contrary, the evolved prefrontal cortex can manage these primitive emotions which will not result in violent action.
(2) At the behavioral level, however, damage to the prefrontal cortex can cause greater impulsiveness, lower perception of risk, and a failure to comply with the rules. These characteristics are widespread among people convicted of violent crimes.
(3) At a personological level, damage of the prefrontal cortex could lead to variations in an individual’s personality. For instance, the famous case study subject Phineas Gage (O’Driscoll and Leach, 1998) had a serious accident in which the prefrontal cortex suffered enormous damage and completely changed his behavior. From a meek and rational man, he became impulsive and violent.
(4) On a social level, damage to the prefrontal cortex can cause an inability to relate to others. One example is the poor social and life skills possessed by some people convicted of violent crimes. Many of them manage stress and anger solely through violent action or aggressive acting-out, not showing problem-solving and decision-making skills.
(5) Finally, on a cognitive level, the prefrontal cortex regulates intellectual flexibility. In fact, by analyzing the school careers of many people convicted of violent crime, it is possible to deduce various intellectual difficulties that often result in violent actions and anger.

Raine (2013) analyzed two examples of the prefrontal cortex’s role in violent behavior. The first one is the story of Antonio Bustamante, a man with a strong bond with his family and who, during a home robbery, killed an elderly a man with his fists, showing a disorganized and chaotic *modus operandi*. At the age of twenty, Bustamante suffered head trauma with a crowbar that caused a change in his personality. Bustamante had transformed from a staid and calm individual to an impulsive and emotionally unstable person. A CT scan showed dysfunction of his prefrontal cortex. After his head trauma, Bustamante was no longer able to have self-control and began to use drugs and commit crimes. Bustamante, together with the damage to the prefrontal cortex – deputy to the decision-making process, behavioral and impulse control, mentalization, and social interactions – also, had injuries to the orbitofrontal cortex, a region associated with automatic discrimination of a stimulus, processing of emotional signals, and affective empathy. These lesions affected his behavioral and emotional control skills: he became more impulsive and unable to reflect on his decisions. This change was also evident in his criminal behavior. Indeed, Bustamante committed an impulsive, unplanned, and disorganized crime, showing reactive violence, a poor capacity to plan and to reflect on the process and the consequences. In fact, Bustamante did not try to erase his tracks and at the time of the arrest, he still had bloodstained clothes on.

The second case is the story of Randy Kraft, a serial killer who killed sixty-four people in 12 years without ever getting arrested. Kraft planned the murders extensively, measuring his actions, predicting, considering alternative plans, and maintaining very high concentration to perform complex tasks. Randy Kraft could represent a psychopath serial killer with high capacity in planning and organization, who commits the so-called “cold blood crimes” (Woodworth and Porter, 2002). Kraft used instrumental violence, purposeful and predatory. His prefrontal cortex was hyper-activated, showing the key role of this region in his capacity to manage social behavior, reduce impulsivity, devise a plan not to get arrested and adapt his conduct according to the context, as shown by studies which demonstrate the role of PFC in impulse and behavioral control, decision-making process and planning (Miller and Cohen, 2001; Spinella, 2004; Palijan et al., 2010; Soyoun and Daeyeol, 2011; Boduszek et al., 2017).

Our brains develop and change in relation and response to the environment, family, and experiences in life, so it is always essential to contextualize the crime and humanize the people in question as well through a deeper analysis which considers social, educational values and environmental and family factors.

## Aims and Procedure

We conducted a review of the literature on and related to empathy, neuroscience, and violent crimes. Electronic databases utilized included: Columbia Libraries Online Catalog, Scopus, PubMed. This review aims to examine the current knowledge on the relationship between empathy, neurological substrates, violent crime, and psychopathy. Specifically, we extended our research of the literature to a less investigated target (woman and youths), often overlooked as they are less likely to commit violent crimes.

We utilized the following search terms: “empathy,” “neuroscience,” “violent,” “psychopath^∗^,” “crim^∗^,” “offend^∗^,” “female,” “child^∗^,” “juvenile,” and “male.” Of the articles returned from the search, eight were retained for the current review after screening their titles and abstracts, as reported in Table 1. The inclusion criteria were as follows: (1) articles on empathy or psychopathy, neuroscience, and violent crimes; (2) articles focused on a currently incarcerated population (male, female, adults, and juveniles); (3) original articles written in English; and (4) articles published in peer-reviewed journals between 2017 and 2021.

**TABLE 1:** Authors, year of publication, subjects, and methods of articles selected for the review.

Authors and year of publication	Population of focus	Method/target
Aghajani et al., 2017	Severely antisocial, conduct-disordered male juvenile offenders convicted of violent crimes	MRI for intrinsic functional connectivity analysis of amygdala
Keune et al., 2017	Adult incarcerated males convicted of violent crime in German high security prison	Resting-state EEG recording of frontal cortex
Sajous-Turner et al., 2019	Adult male participants from prisons in New Mexico and Wisconsin (homicide, violent non-homicide, non-violent)	MRI for voxel based morphometric analysis of gray matter
Hofhansel et al., 2020	Adult incarcerated males convicted of violent crime in German high security prison	MRI for voxel based morphometric analysis of gray matter
Vermeij et al., 2018	Adult males placed in Pieter Baan Center (Netherlands) for forensic psychiatric evaluation	Diffusion-weighted MRI of white matter
Raine, 2018	Juveniles and adults with antisocial, violent, and psychopathic behavior	Review: update of neuromoral theory of impairment to neural circuitry in antisocial behaviors
Calzada-Reyes et al., 2020	Adult males and females with psychopathy, incarcerated in Cuba for violent criminal acts	Quantitative EEG, low-resolution electromagnetic tomography (LORETA) to assess electrophysiological sex-influenced differences

Articles published in a language other than English, duplicate articles and articles published before 2017 were excluded from the review.

## Principal Findings

The selected studies analyzed the connection between psychopathic or callous-unemotional (CU) traits and brain abnormalities in people convicted of violent crimes. However, these studies are heterogeneous by sample and research methodology with implications for the generalizability of the results.

A study conducted in 2017 (Aghajani et al., 2017) probed the intrinsic functional connectivity of amygdala networks across a healthy control group and two groups of male juveniles (15–19 years old) clinically diagnosed as conduct-disordered (CD) and convicted of a violent crime: those with CU traits (CD/CU+) and those without CU traits (CD/CU-). Aiming to understand how subregional amygdala connectivity might contribute to callous-unemotionality in conduct-disordered youth, Aghajani et al. (2017) focused on the basolateral amygdala (BLA) and centromedial amygdala (CMA) complexes. The BLA is heavily involved in integrating affective value for incoming emotionally salient stimuli (Sah et al., 2003), while the CMA serves as the primary site for efferent signals from the amygdala, directing physiological and behavioral responses to emotional stimuli (LeDoux, 2007).

Upon collecting magnetic resonance imaging (MRI) data and analyzing functional connectivity for the three groups of juveniles, the researchers found that CD/CU+ youths had increased right BLA connectivity and decreased left CMA connectivity, including the vmPFC. Additionally, they found that CD/CU+ youth had lower mean bihemispheric amygdalar volumes relative to healthy controls due to hypotrophy of BLA and CMA subregions. These findings show that CD youth with CU traits and convicted of violent crimes have abnormal amygdalar connectivity and volumes in areas consistently implicated in psychopathy.

In another study conducted with adult males in a high security prison convicted of violent offences (Keune et al., 2017), the researchers utilized EEG to measure alpha wave asymmetry, a phenotypic indicator of approach vs. withdrawal behavior patterns, in the frontal cortex (Harmon-Jones et al., 2010). The approach-withdrawal model has linked anger and aggression to an approach pattern and higher relative anterior cortical activity in the left hemisphere (Peterson et al., 2008). Therefore, the researchers hypothesized that CU traits would be associated with approach-related patterns connected to aggression. However, they ended up finding that CU traits were associated with stronger relative anterior cortical activity in the right hemisphere (i.e., withdrawal-related patterns) for the males convicted of violent crimes. This suggests that callousness may be related to withdrawal despite its connection with aggressive and violent behavior.

Another study from 2019 (Sajous-Turner et al., 2019) reported findings on gray matter volume in three groups of adult incarcerated males: those who had committed homicide, those who had committed violent crimes but not homicide, and those who had committed minimally violent or non-violent crimes. MRI scans and subsequent statistical analysis revealed that males convicted of violent crimes had large deficits in the orbitofrontal/ventromedial prefrontal cortex, anterior temporal cortex, insula, medial prefrontal/anterior cingulate and precuneus/posterior cingulate cortex compared to males convicted of non-homicidal and minimally violent crimes. Because these regions have been notably implicated in empathy and general emotional processing (Decety, 2011), these findings provide insight into how abnormalities in regions for social cognition may distinguish the brains of those who commit homicide from those who commit other types of crimes.

A similar study (Hofhansel et al., 2020) focusing on gray matter volume was undertaken with adult incarcerated males convicted of at least one violent crime and a control group, aiming to elucidate specific brain morphology to both reactive aggression and psychopathy. MRI data revealed that increased PCL-R sum scores correlated with decreased gray matter volume in the superior prefrontal cortex, confirming the previous literature linking global psychopathy to reductions in prefrontal gray matter (Pujol et al., 2018). Going further, however, the researchers found that this correlation was primarily driven by the subscale of the PCL-R score related to antisocial behavior, particularly for gray matter reductions in the right superior frontal and left inferior parietal regions. Additionally, decreased gray matter volume in the right middle and superior temporal gyrus were correlated with both reactive aggression and antisocial behavior. With these findings, the researchers suggested that the volume of brain regions involved in ToM (i.e., the ability to understand the beliefs and intentions of others) are reduced in antisocial males convicted of a violent crime.

Vermeij et al. (2018) compared white matter variations in relation to psychopathic traits between incarcerated males with impulse control problems and incarcerated males without impulse control problems. Upon analysis of diffusion-weighted MRI data, interpersonal-affective traits (PCL-R Factor 1) were found to be inversely correlated with white matter integrity in the anterior and posterior temporal lobe and orbitofrontal area in impulsive males. More specifically, increased affective traits (PCL-R Facet 2) were associated with reductions in white matter integrity in the right temporal lobe. Importantly, these findings link disrupted neural connectivity with affective psychopathic deficits specifically in impulsive incarcerated males, refining the associations of brain morphologies to different facets of psychopathy.

In 2018, Raine probed the neuromoral theory of antisocial, violent, and psychopathic behavior. The author noted that the existing model proposes an overlap in many of the brain regions and mechanisms involved in both antisocial or psychopathic behavior and moral decision-making. Raine verified and revised this model with new empirical findings from the literature. Overall, most individuals convicted of crimes are predicted by the model to have neuromoral impairment in the fronto-polar, medial prefrontal, anterior cingulate, insula, superior temporal gyrus, amygdala, and angular gyrus to some degree. Primary psychopathy is characterized by the core psychopathic features, while secondary psychopathy is characterized more by increased impulsivity and reactive aggression. The neuromoral model predicts that stronger neuromoral impairment is linked more to primary psychopathy and weaker neuromoral impairment is linked to the secondary psychopathy. Raine (Ibidem) showed that this distinction has large implications for violent crime. Indeed, stronger neuromoral impairment is connected to more proactive aggression that is described as predatory and more planned, while milder neuromoral impairment is connected to more reactive aggression that is linked to decreased emotional and impulse control.

There is little existing research that assesses the neurobiological correlates of empathy among psychopathic female or offenders for violent crime. A singular study incorporated females by investigating gender differences in electrophysiology in people with psychopathy and convicted of a violent crime (Calzada-Reyes et al., 2020). Resting EEG visual analyses revealed that both females and males had a high percentage of EEG abnormalities compared to normative database values. The researchers noted specific differences between psychopathic males and females in brain connections and regions that regulate emotion, decision making, and moral judgment. These regions include the bilateral frontal and centroparietal areas, parieto-occipital areas, and the basal ganglia. These findings show that there are similar frequencies of EEG abnormalities in psychopathic males and females, but that there are still significant differences between both groups that may prove beneficial when differentiating and screening the two.

## Discussion and Conclusion

The analysis of the cited literature shows the role that empathy has, both on an emotional and cognitive level, (Saladino et al., 2020a) in violent and psychopathic behavior (Nascivera et al., 2019). Empathy aims to guide an individual’s behaviors according to the understanding of others’ emotional states. In recent years, psychologists and neuroscientists have studied the possible association between empathic deficit, neuroscience, violent crime, and psychopathic personality.

Hare (1991) and Baron-Cohen (2011) were two scientists who studied the construct of psychopathy. Hare classified this concept, structuring a questionnaire to evaluate it, the Psychopathy Checklist-Revised (PCL-R) (Hare, 2003). This questionnaire was used by the author and other researchers among the US prison population, where subsequent results reported a high level of psychopathy diagnosis among violent offenders (Ibidem; Coid et al., 2009; Lavazza and Sammicheli, 2012; León-Mayer et al., 2015).

Simon Baron-Cohen (2011) associated psychopathy with a low level of empathy, assuming that empathy is divided into seven grades and psychopaths are affected by an empathic defect and have zero grade of empathy because of it.

Neuroscientific evidence (Gallese and Goldman, 1998; Preston and de Waal, 2002) identified a core empathy network, which involve several areas of the brain, such as AI, the posterior-anterior, and the anterior medial cingulate cortex, involved in pain processing, perception of emotions, and interoceptive awareness (Kober et al., 2008; Craig, 2009; Duerden and Albanese, 2011; Lindquist et al., 2012); the vmPF, the superior temporal sulcus, the temporoparietal junction, and the posterior cingulate cortex, associated with ToM and mentalization ability; the dorsolateral PFC and dorsomedial PFC, and the inferior parietal cortex, deputy to the perception-based condition of empathy (Danziger et al., 2009; Zaki et al., 2009). Furthermore, the affective empathy is regulated by the amygdala, hypothalamus, and orbitofrontal cortex for the affective arousal; while the cognitive empathy is associated with anterior insula, medial prefrontal cortex, and ventromedial prefrontal cortex for emotion awareness, and OFC, mPFC, and dorsolateral prefrontal cortex for emotion regulation, control of emotion and motivation.

Other studies found a reduction of gray matter in the so-called “moral brain” that can lead to immoral decisions (De Oliveira-Souza et al., 2008); an emotional deficit in the “emotional brain,” involved in the management of emotions (Kiehl et al., 2001; Kiehl, 2006); and a social deficit in the “social brain” that can compromise facial expressions’ processing (Malatesti and McMillan, 2010).

In his studies, Raine (2013) demonstrated and confirmed the role of the prefrontal cortex in violent and psychopathic behaviors. The author identified five levels of influence of the prefrontal cortex in violence: emotional, behavioral, personological, social level, and cognitive level. Also, the author described two different aggressive behaviors and attitudes that characterized different offenders. He showed two examples of the prefrontal cortex’s activation in two violent killers. The first one, Antonio Bustamante, disorganized and impulsive, committed homicide during a robbery; the second, Randy Kraft, always planning and organizing actions, committed sixty-four murders. From the CT scan, Bustamante showed dysfunction of the prefrontal cortex related to a head trauma caused by an accident during his youth. On the contrary, Kraft presented with a hyper-activation of the prefrontal cortex. These results explained Bustamante’s impulsiveness and poor self-control and Kraft’s organization and meticulousness. Indeed, the damage to both PFC and OFC affected Bustamante’s capacity of decision-making and self-control. Bustamante acted using reactive aggression without mentalization of his actions and the related consequences, showing a deficit in affective empathy. Damage of the PFC is known to be associated with an increased impulsivity and a decreased decision-making (Miller and Cohen, 2001; Spinella, 2004; Palijan et al., 2010; Soyoun and Daeyeol, 2011; Boduszek et al., 2017). On the other hand, Randy Kraft had a hyperactivation of PFC which allowed him to plan his murders for years. He represents a psychopath who act cold blood crimes, using instrumental violence. In this case, the role of PFC was related to the management of social behavior, the reduction of impulsivity, and the adaptability to the context. The two reported cases represent two opposite ways of acting criminal behavior and highlight the role of the PFC in violent actions. Moreover, as reported by Boduszek et al. (2017), the investigation of the IQ related to empathy, psychopathy, and violent crimes is needed. Indeed, according to the authors, IQ can moderate the relations between psychopathy and the ability to emotionally respond. Future research could focus on this connection to better understand the role of IQ in empathy and psychopathy.

Furthermore, several studies have focused on lesions and damage of brain areas involved in the core network of empathy, while less is known about the hyperactivation and the higher functioning of some areas, as PFC, OFC, or NAcc in subjects with psychopathic traits or violent offenders.

The reviewed literature converges on the fact that psychopathy, the lack of empathy, and violent crime relate to abnormalities in brain morphology, connectivity, or activity. However, the targets and methods used in each study were varied. Of the seven empirical studies reviewed, two (Sajous-Turner et al., 2019; Hofhansel et al., 2020) used the same method: MRI for voxel based morphometric analysis of gray matter volume. Both studies confirm that adult males convicted of violent crimes had gray matter reductions in the prefrontal cortex and temporal gyrus, areas consistently implicated in emotional control, antisocial behavior, and reactive aggression. Two other studies also utilized the same method: EEG (Keune et al., 2017; Calzada-Reyes et al., 2020). However, Keune et al. (2017) focused on alpha wave asymmetry to probe approach vs. withdrawal behavior, while Calzada-Reyes et al. (2020) focused on beta activity to analyze differences between psychopathic females and males in terms of excitability or arousal.

The other three studies chose different methods of evaluation. Vermeij et al. (2018) measured connectivity via diffusion tensor imaging of white matter tracts across the whole brain, Aghajani et al. (2017) measured connectivity via MRI in the amygdala specifically. Jones et al. (2018) utilized fMRI to investigate neural correlates of empathy.

In most of the studies, the same brain regions are consistently implicated in relation to CU traits, aggression, or psychopathy: the prefrontal cortex, amygdala, and temporal cortex. As discussed before, the PFC has a key role in emotional and behavioral control, especially over the limbic system, which is related with primary emotions like anger and fear. The PFC is also related to social behavior and adaption to different context. People who suffer from damage in this area can behave aggressively, show poor problem-solving and decision-making skills, or change personality. At the same time, people who have a high level of activation in this area can present some traits of primary psychopathy, such as manipulation, self-overestimation, instrumental violence, pathological lying, superficial charm, lack of emotions, low levels of empathy, and remorse. These subjects can conduct a normal life or commit crimes without being involved in the criminal justice system because they present a strong capacity to adapt their personality to others and social needs, protecting themselves from being noticed.

The amygdala is fundamental in affective empathy and emotional arousal (Hurlemann et al., 2010) and in the perception of external stimuli. Impulsive aggression could derive from an activation of motoric aggressive responses in absence of control by the OFC and the anterior cingulate cortex, which regulate the social behavior according to the reward and punishment expectations. These regions can repress aggressive behavior when individuals perceive negative consequences through the limbic regions, such as the amygdala and insula (Siever, 2008). Stimuli can become triggers for violent behavior due to a perceptive distortion in sensory processing centers. These distortions can derive from alcohol, substance use, or illness and psychopathologies, leading someone to perceive a stimulus as provocative or dangerous and to react aggressively. These stimuli are processed to higher levels from the prefrontal, temporal, and parietal cortices. Then, the processed information can be filtered according to the sociocultural values and experiences of the person (aspects related to the amygdala and limbic regions). Furthermore, there are psychopathologies that, together with negative experiences, can create the condition that leads someone to interpret stimuli as aggressive and respond with violence. For instance, fMRI studies demonstrated the influence of personality disorder and psychopathologies in the perception of external stimuli. Participants with borderline or antisocial personality disorder, characterized by a lack of impulse control and a tendency to be aggressive, perceived anger when evaluating emotional expressions of others (Best et al., 2002; Coccaro et al., 2007); on the other hand, people with anxiety disorder mostly identified fear in facial expressions.

The temporal cortex is involved in moral decisions (De Oliveira-Souza et al., 2008). Mostly, patients with tumors or epilepsy in temporal lobe or with temporal lesions report highly aggressive behavior (Tonkonogy and Geller, 1992; Ito et al., 2007). Moreover, structural alterations in the temporal cortex, together with the medial temporal cortex and hippocampus, are common among people with antisocial personality disorder (Raine et al., 2004).

The role of impairment in function or morphology of the cited regions is supported by converging evidence of the cited literature through different methods of evaluation.

The interplay between CU traits and proactive vs. reactive aggression needs to be further studied. According to the EEG data and the predictions of the neuromoral model (Keune et al., 2017; Raine, 2018), the consensus seems to be that callousness is related to more reactive aggression or withdrawal behavior. However, the implications for the degree of violence in crime committed are still unclear.

Despite several studies on gender difference in neuroanatomy and neuronal structure, as well as the USA National Institute of Mental Health’s incorporation of gender as a variable of influence in neurological and psychiatric studies (National Institute of Mental Health, 2011; Zagni et al., 2016), little is known on the role that these differences could have in the association between empathy, violent crime, and psychopathy. Indeed, male populations, specifically offenders, receive greater attention than females. Some of the main reasons are that men represent a large part of the prison population, are diagnosed psychopaths more than women, and females tend to be more empathic and emotionally sensitive than men (Edwards et al., 2019b; Calzada-Reyes et al., 2020). It is therefore more difficult to find a sample of female prisoners who have committed violent crimes and who can be compared with males. Although a few previous studies and reviews have focused on psychopathy among incarcerated females (Hornsveld et al., 2018; Edwards et al., 2019a; Thomson et al., 2019), they have not specifically probed the neurobiological correlates of psychopathy in relation to violent crimes. The lone study in this review that included females (Calzada-Reyes et al., 2020) is a step toward uncovering more knowledge about individual risk factors and significant trends based on subsets of the population. They reported that there are differences between psychopathic males and females in brain connections and regions, specifically bilateral frontal and centroparietal areas, parieto-occipital areas, and the basal ganglia, involved in emotion regulation, decision-making process, and moral judgment. However, there are similar frequencies of EEG abnormalities in both samples. By expanding the breadth of research in terms of gender and age, the generalizability of these results can be more readily evaluated.

Lastly, the need for interdisciplinary research on this complicated topic at the intersection of sociology and neuroscience is pressing. Social conditions influence the level of engagement in crime, the development of psychopathologies, or personality disorders related to crime. For instance, criminogenic or poor environments could increase exposure to drugs, violence, and dysfunctional behaviors, which, especially during childhood and adolescence, can develop in antisocial personality disorder, psychopathy, and other related issues (Saladino et al., 2020c). Additionally, the development of empathic abilities derives also from daily learning and from exposure to positive and prosocial environments (Lamm et al., 2011). The parent-children’s attachment, the parenting and the communication style, and the family climate affect the level of emotional regulation, the processing of emotions, and the ability to understand and react to social stimuli (Saladino et al., 2020b). In fact, research in the field of developmental psychology and social psychology shows how neglect, violence, poor communication, and inadequate education can affect the socialization and development of the child (Hetherington, 1987; Massarwi and Khoury-Kassabri, 2017). As illustrated by the multifactorial theories on crime, it is not possible to give a univocal explanation for criminal behavior, but all the co-necessary factors must be evaluated. One of the main limits is certainly connected to the fragmentation of knowledge and the separation of the psychological, sociological, and neuroscientific side, which should instead collaborate to give a more effective and truthful vision of the criminogenic phenomenon and its neuro-sociological implications (Auriemma et al., 2020).

As Raine (2018) mentions in his review, one of the biggest weaknesses of the current neuromoral theory is its lack of incorporation of social circumstances. The heterogeneity of violent behavior and crimes exhibited by the incarcerated individuals studied makes it difficult to match and include a control community sample, as one study found (Sajous-Turner et al., 2019). Additionally, the studies reviewed cannot account for whether the setting in which their subjects are incarcerated influences the results that are attributed to psychopathy, callous-unemotional traits, and violent behavior (i.e., whether institutionalization causes violence and exacerbates psychopathic traits or vice versa).

By not accounting for policing and sentencing biases, the varying definitions of crime, and the complexities of the criminal legal system, neuroscientific research by itself cannot provide an accurate picture of violent crime, a multi-faceted issue that must consider the social world.

## Author Contributions

VS, HL, EZ, and VV conceptualized the contribution. VS, EZ, and HL wrote the manuscript. VV reviewed the manuscript. All authors approved the submission of the manuscript.

## Conflict of Interest

The authors declare that the research was conducted in the absence of any commercial or financial relationships that could be construed as a potential conflict of interest.

## Publisher’s Note

All claims expressed in this article are solely those of the authors and do not necessarily represent those of their affiliated organizations, or those of the publisher, the editors and the reviewers. Any product that may be evaluated in this article, or claim that may be made by its manufacturer, is not guaranteed or endorsed by the publisher.

## Abbreviations

AI: anterior insula
aMCC: anterior medial cingulate cortex
ASN: autonomic nervous system
BLA: basolateral amygdala
CD: conduct-disordered
CMA: centromedial amygdala
CU: callous-unemotional
dlPFC: dorsolateral prefrontal cortex
dmPFC: dorsomedial prefrontal cortex
EEG: electroencephalogram
EQ: empathy quotient
fMRI: functional magnetic resonance imaging
FTD: frontotemporal dementia
HD: Huntington’s disease
HPA: hypothalamic-pituitary-adrenal axis
IPC: inferior parietal cortex
IQ: intelligence quotient
MCC: middle cingulate cortex
mPFC: medial prefrontal cortex
MRI: magnetic resonance imaging
NAcc: nucleus accumbens
OFC: orbitofrontal cortex
pACC: posterior anterior
PCC: posterior cingulate cortex
PCL-R: Psychopathy Checklist-Revised
PET: positron emission tomography
PFC: prefrontal cortex
PPTM: Psychopathic Personality Traits Model
STS: superior temporal sulcus
ToM: theory of mind
TPJ: temporoparietal junction
vmPFC: ventromedial prefrontal cortex.
